# Antimicrobial Usage, Susceptibility Profiles, and Resistance Genes in *Campylobacter* Isolated from Cattle, Chicken, and Water Samples in Kajiado County, Kenya

**DOI:** 10.1155/2023/8394605

**Published:** 2023-03-22

**Authors:** Daniel W. Wanja, Paul G. Mbuthia, Lilly C. Bebora, Gabriel O. Aboge, Brian Ogoti

**Affiliations:** ^1^University of Nairobi, Faculty of Veterinary Medicine, Department of Veterinary Pathology, Microbiology and Parasitology, P.O. Box 29053, Kangemi, 00625 Nairobi, Kenya; ^2^Animal Health and Industry Training Institute (AHITI) Kabete, P.O. Box 29040, Kangemi, 00625 Nairobi, Kenya; ^3^Department of Animal Science, Chuka University, P.O. Box 109, 00625 Chuka, Kenya; ^4^University of Nairobi, Faculty of Veterinary Medicine, Department of Public Health, Pharmacology and Toxicology, P.O. Box 29053, Kangemi, 00625 Nairobi, Kenya; ^5^University of Nairobi, Faculty of Health Sciences, Center for Epidemiological Modelling and Analysis, Kenyatta National Hospital Nairobi, P.O. Box 19676, 00202 Nairobi, Kenya

## Abstract

*Campylobacter* organisms are the major cause of bacterial gastroenteritis and diarrhoeal illness in man and livestock. *Campylobacter* is growingly becoming resistant to critically crucial antibiotics; thereby presenting public health challenge. This study aimed at establishing antimicrobial use, susceptibility profiles, and resistance genes in *Campylobacter* isolates recovered from chicken, cattle, and cattle-trough water samples. The study was conducted between October 2020 and May 2022 and involved the revival of cryopreserved *Campylobacter* isolates confirmed by PCR from a previous prevalence study in Kajiado County, Kenya. Data on antimicrobial use and animal health-seeking behaviour among livestock owners (from the same farms where sampling was done for the prevalence study) were collected through interview using a pretested semistructured questionnaire. One hundred and three isolates (29 *C. coli* (16 cattle isolates, 9 chicken isolates, and 4 water isolates) and 74 *C. jejuni* (38 cattle isolates, 30 chicken isolates, and 6 water isolates)) were assayed for phenotypic antibiotic susceptibility profile using the Kirby–Bauer disk diffusion method for ampicillin (AX), tetracycline (TE), gentamicin (GEN), erythromycin (E), ciprofloxacin (CIP), and nalidixic acid (NA). Furthermore, detection of genes conferring resistance to tetracyclines (*tet* (O), *β*-lactams (*bla*_*OXA*-61_), aminoglycosides (*aph*-3-1), (fluoro)quinolones (*gyrA*), and multidrug efflux pump (*cmeB*) encoding resistance to multiple antibiotics was detected by mPCR and confirmed by DNA sequencing. The correlation between antibiotic use and resistance phenotypes was determined using the Pearson's correlation coefficient (*r*) method. Tetracyclines, aminoglycosides, and *β*-lactam-based antibiotics were the most commonly used antimicrobials; with most farms generally reported using antimicrobials in chicken production systems than in cattle. The highest resistance amongst isolates was recorded in ampicillin (100%), followed by tetracycline (97.1%), erythromycin (75.7%), and ciprofloxacin (63.1%). Multidrug resistance (MDR) profile was observed in 99 of 103 (96.1%) isolates; with all the *Campylobacter coli* isolates displaying MDR. All chicken isolates (39/39, 100%) exhibited multidrug resistance. The AX-TE-E-CIP was the most common MDR pattern at 29.1%. The antibiotic resistance genes were detected as follows: *tet* (O), *gyrA*, *cmeB*, *bla*_*OXA-61*_, and *aph*-3-1 genes were detected at 93.2%, 61.2%, 54.4%, 36.9%, and 22.3% of all *Campylobacter* isolates, respectively. The highest correlations were found between *tet* (O) and tetracycline-resistant phenotypes for *C. coli* (96.4%) and *C. jejuni* (95.8%). A moderate level of concordance was observed between the Kirby–Bauer disk diffusion method (phenotypic assay) and PCR (genotypic assay) for tetracycline in both *C. coli* (kappa coefficient = 0.65) and *C. jejuni* (kappa coefficient = 0.55). The study discloses relatively high resistance profiles and multidrug resistance to antibiotics of critical importance in humans. The evolution of the multidrug-resistant*Campylobacter* isolates has been linked to the use and misuse of antimicrobials. This poses a potential hazard to public and animal health, necessitating need to reduce the use of antibiotics in livestock husbandry practice coupled with stringent biosecurity measures to mitigate antimicrobial resistance.

## 1. Introduction

Campylobacters are widely distributed as a normal flora in the gut of both domestic and wild animals, and are also found in environmental samples, including surface water, soil, and feeds [1]. The incidence of campylobacters in environmental sources is mainly related to fecal contamination. Poultry are the main reservoirs though bovine, swine, shoats, dog, and cat have also been recognized as other probable reservoirs for human disease. Cattle-derived isolates can infect poultry painting a picture that cattle could be a source of infections to chicken [2], and vice versa.

Of the more than 25 species in the genus *Campylobacter*, *C. jejuni* and *C. coli* have been reported to cause major public health burden globally. *Campylobacter jejuni* and *C. coli* accounted for 98% of cases reported in humans in 2015–2017 in the USA [3], with the WHO projecting that *Campylobacter* causes 37,600 fatalities/year globally [4]. This burden is even higher than the burden caused by salmonellosis [5]. *Campylobacter* is the major cause of food-borne infections in man through ingestion of raw and/or poorly cooked contaminated food of animal origin (be it beef, pork, or chicken meat), and consumption of contaminated water and raw milk. In addition, cross-contamination of fast foods during preparation, besides coming into contact with faeces from sick humans and companion animals has also been reported as risk factors [6, 7]. *Campylobacter* illness in humans manifests itself as episodes of gastroenteritis accompanied by abdominal pain, biliousness, unsettled stomach, pyrexia, and watery diarrhoea and/or dysentery [8]. However, in infants and in patients with lowered immunity, *Campylobacter jejuni* is associated with postinfection sequelae including Guillain-Barre syndrome and/or Miller Fischer Syndrome (demyelinating neuropathies affecting peripheral nerves), reactive arthritis, meningitis, myocarditis [9], and fatal septicaemic infection.

Although *Campylobacter* infections in humans are sporadic and often self-limiting, antimicrobial therapy is indicated in severe and prolonged cases of enteritis, immunosuppressed individuals, and/or in young children. Macrolides (erythromycin) and fluoroquinolones (FQs) (ciprofloxacin) are considered the last resort drugs in clinical cases requiring therapy. However, other classes of antibiotics including aminoglycosides (gentamicin), tetracyclines, lincosamide (clindamycin), and penicillin (ampicillin) can be prescribed as substitute medication for the management of septicaemic campylobacteriosis. However, over the decades, several studies in Kenya and beyond, have reported an increase in infections caused by multidrug-resistant (MDR) *Campylobacter* [10–12].

There is little information on antibiotic susceptibility profiles of *Campylobacter* strains emanating from food animals in Kenya; and even then, the few available studies are in humans. In addition, no previous studies have been conducted in Kajiado County on antibiograms of thermotolerant *Campylobacter* species from food animals, despite the high dependency and/or consumption of animal protein in this county. The few animal-based studies conducted in other regions in Kenya have only focused on resistance profiles displayed by chicken *Campylobacter* isolates [12–14], without investigating the resistance situation in cattle and their respective environment. It is worth noting that, phenotypic antibiotic resistance may be caused by many different genetic determinants which may present particular epidemiological characteristics [15]. Of particular concern are the genetic determinants encoding MDR [16], especially when disseminated with AMR phenotypes. Furthermore, evaluation of genetic determinants of resistance is vital for elucidating and controlling antimicrobial resistance, i.e., it can be used to reliably predict resistant phenotypes. Therefore, it is of paramount importance to delve into the genetic mechanisms linked to antibiotic resistance in *Campylobacter* species. The genetic determinants of antimicrobial resistance in *Campylobacter* have been characterized exquisitely in studies conducted in other countries. They showed that resistance genetic determinants in *Campylobacter* are mediated by the following: (1) existence of *tet* (O), *tet* (M), and/or *tet* (A) genes which are responsible for resistance against tetracyclines [17]; (2) point mutations in the *gyrA* and 23S rRNA genes which contributes to FQs and macrolide resistance, respectively; (3) an efflux pump (*cme*ABC) which reduces the intracellular concentration of antimicrobials; works synergistically with other resistance mechanisms and contributes to the resistance to multiple antibiotics; and (4) presence of “naturally” occurring resistance genes against *β*-lactams (e.g., ampicillin), mainly due to the ubiquitousness of the *bla*_OXA-61_ gene [18, 19]. In addition, alleles of other genes associated with resistance to aminoglycoside (e.g., aminoglycoside 3′-phosphotransferase gene (*aph*-3-1)) have also been reported [20].

The emergence and spread of AMR among *Campylobacter* spp. in the livestock sector and human health contexts have been linked to the overuse or inappropriate usage of antimicrobial drugs. Antimicrobials are used to treat sick animals (therapeutic purposes), prevent livestock diseases (both prophylactic and metaphylactic purposes), and enhance growth. However, any application of antimicrobials, whether considered curative or not, deliberate or otherwise, exposes both pathogenic bacteria and gut commensals to varying concentrations for varying times [21]. This creates a selective pressure that can result in evolution and spread of resistance or an increase in the abundance of resistant bacteria, especially where a resistant subpopulation exists [21]. As such, there is an urgency to control antimicrobial resistance (AMR) amid the rampant failure in veterinary and/or human medicines. The scourge of antimicrobial resistance in Kajiado and Kenya at large is further compounded by the collapse of public services in the 1980s, including veterinary services. With privatization of veterinary services, delivery of animal health services, more so in arid and semiarid countries, has become a nightmare. Alternatives to this new reality include engaging “community-based animal health workers” (CAHWs) in treating animals [22]. CAHWs lack continuous training on/or up-to-date know-how on antimicrobial use (AMU) and treatment guidelines, and may end up prescribing inappropriate antimicrobial therapy, including the controlled antimicrobials for humans and animals. While in some developed countries including Australia and Korea; use of fluoroquinolones (FQs) and gentamicin in livestock including poultry was banned over a decade ago [20, 23]; the same antibiotics continue to be used in livestock in Kenya. Furthermore, Kajiado County is dominated by the Maasai, one of Kenya's major pastoralists, who are known to self-treat and/or engage unskilled people to treat their sick animals with antibiotics. Here, the resistance begins. As such, there is a need to monitor antimicrobial use (AMU) practice, so as to minimize the development of AMR. However, significant knowledge gaps exist on the exact quantities, frequency, and types of antimicrobials being used in cattle and chicken production systems at farm level in Kajiado and Kenya at large.

As a result of the widespread resistance to multiple antibiotic classes, it is no surprise that the World Health Organization has listed fluoroquinolone-resistant *Campylobacter* as a high priority pathogen; with the objective of more research and development of new antibiotics [24]. In the wake of these glaring realities and scarce published data on AMU and AMR in Kenya, this study aimed to investigate antimicrobial use, susceptibility profiles, and resistance genes in *Campylobacter* isolates from chicken, cattle, and water in Kajiado County, Kenya.

## 2. Materials and Methods

### 2.1. Ethical Consideration

The Biosafety, Animal Use, and Ethics Committee of the Faculty of Veterinary Medicine, University of Nairobi, approved this study under the reference: FVM BAUEC/2020/274. Verbal consent was sought from farm owners prior to interviewing.

### 2.2. Study Area, Design, and Selection of Production Systems

**Figure 1:**
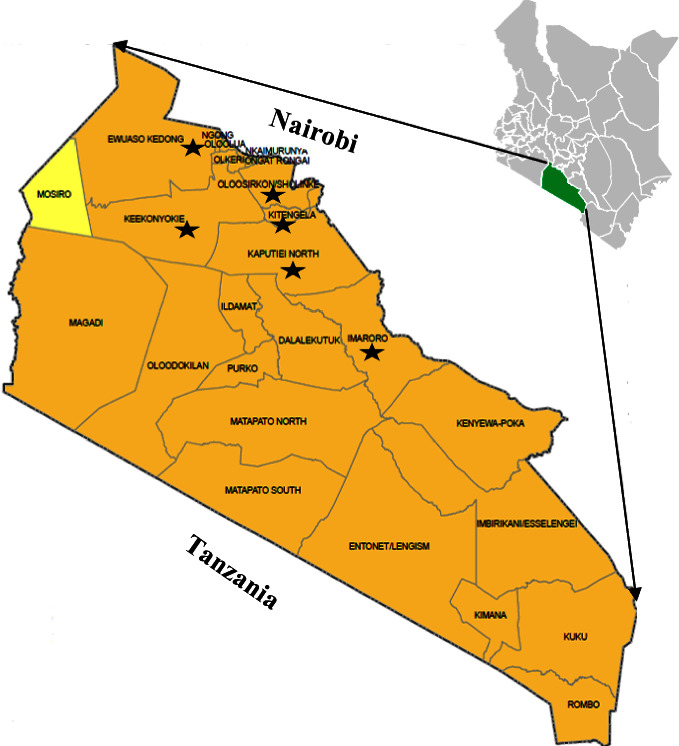
A map of Kajiado County showing its location in Kenya and sites where sampling and interviews on antimicrobial use among livestock farmers were conducted.

A field and laboratory-based cross-sectional study design was conducted between October 2020 and May 2022 in Kajiado County, located south of Nairobi, Kenya (Figure 1). The county has well-established smallholder mixed-livestock (cattle and poultry) production systems. These production systems were chosen based on the fact that (1) poultry production is the highest consumer of antimicrobials; (2) there is sketchy information on antimicrobial use in cattle production systems and environmental samples (water).

### 2.3. Origin of *Campylobacter* Isolates

*Campylobacter* isolates used in this study were obtained from a previous study on seasonal prevalence of thermophilic *Campylobacter* from chicken cloacal swabs, cattle rectal swabs, and water samples from cattle-troughs in Kajiado County, Kenya [25]. These isolates were cryopreserved in pure colonies in tryptone soya broth (Hi-media) with 30% glycerol and in the respective genomic DNA in a deep freezer at −20°C. In this study, 119 *Campylobacter* species (29 *C. coli* (16 cattle isolates, 9 chicken isolates, and 4 isolates from water samples isolates) and 90 *C. jejuni* (42 isolates from bovine, 42 isolates from chicken, and 6 water isolates)) from the prevalence study were used.

### 2.4. Survey on Antibiotic Use (AMU) and AMR Awareness

Data on antimicrobial use were collected through administration of semistructured questionnaire in the same farms where sampling was done for the prevalence study. Farm owners/respondents were requested to avail any drugs or used drug containers/sachets kept at the house/farm; these were then recorded accordingly. In farms that indicated to have used antibiotics but had disposed of the container/sachet, the respondents were asked if they could recall the drugs used by their trade name. The survey also concentrated on local disease histories, animal health-seeking behaviours, and AMR awareness.

### 2.5. Phenotypic Antibiotic Susceptibility Profile Using Kirby–Bauer Diffusion Method

The antimicrobial susceptibility of *C*. *jejuni* and *C*. *coli* isolates was established using the Kirby–Bauer disc diffusion technique on plates containing Mueller–Hinton agar augmented with 10% defibrinated ovine blood (MHBA): strictly in accordance with the procedures of the Clinical and Laboratory Standards Institute (CLSI) [26]. Standard antimicrobial impregnated disks (HiMedia Mumbai, India) containing 6 different antibiotics at the given concentration were used as follows: (1) 25 *μ*g ampicillin (AMP); (2) 10 *μ*g gentamicin (GEN); (3) 5 *μ*g ciprofloxacin (CIP); (4) 30 *μ*g nalidixic acid (NA); (4) 15 *μ*g erythromycin (E); and (5) 30 *μ*g tetracycline (TE).

PCR-confirmed *C. jejuni* and *C. coli* isolates from cryopreserved stocks in tryptone soya broth (HiMedia) with 30% glycerol were defrosted and then revived by direct plating on blood agar plates augmented with selective supplement (SR0167 E, Oxoid®) and 10% lysed ovine blood. Then, the inoculated plates were incubated for 36 hours at 42°C under microaerobic conditions. Of 119 *Campylobacter* isolates, 103 (29 *C. coli* (16 isolates from cattle, 9 isolates from chicken, and 4 isolates from water samples) and 74 *C. jejuni* (38 isolates from bovine, 30 isolates from chicken, and 6 isolates from water)) were recovered. However, 16 *C. jejuni* isolates (4 from bovine and 12 from chicken) could not be recovered from TSB-glycerol stocks. Colonies of previously revived *Campylobacter* isolates were emulsified in physiological saline and then diluted to a turbidity equivalent to that of the 0.5 McFarland standards. Fresh uninoculated MHBA plates were initially dried in an incubator at 35°C with the lid removed for 15 minutes prior to inoculation. Sterile swabs were then used to seed the suspension onto MHBA plates, to produce confluent growth. The inoculum was allowed to dry for 5 minutes, then, antibiotic discs were placed on the plate. The seeded plates were microaerobically incubated overnight at 42°C. *C*. *coli* (ATCC 33559) and *C. jejuni* (NCTC 11168) were used as positive controls.

The inhibition zone diameters around antibiotic (ciprofloxacin, erythromycin, and tetracycline) discs were measured, recorded, and then construed as sensitive and/or resistant, following [26] breakpoints guidelines for infrequently isolated or fastidious organisms (M45) including *C. jejuni* and *C. coli*. Since CLSI's M45 (third edition) have no interpretive criterion for inhibition diameters for ampicillin, nalidixic acid, and gentamicin for *C. jejuni* and *C. coli*, the breakpoints provided by CLSI [27], (M100S) for the *Enterobacteriaceae* family was used instead.

### 2.6. Detection of Genes Conferring Resistance to Antibiotics

Genomic DNA of 103 *Campylobacter* isolates (29 *C. coli* (16 isolates from cattle, 9 isolates from chicken, and 4 isolates from water samples) and 74 *C. jejuni* (38 isolates from bovine, 30 isolates from chicken, and 6 isolates from water)) were screened for five genes conferring antimicrobial resistance as follows: multidrug efflux pump *cmeB* gene, aminoglycoside 3′-phosphotransferase gene *aph-3-1* gene, tetracycline resistance *tet*(O) gene, ampicillin (*bla*_OXA-61_) gene, and quinolone resistance topoisomerase gene (*gyrA*). The forward (F) and reverse (R) primers specific for the antibiotic resistance genes used in this study were designed based on the gene sequences of previously published studies: *tet*O-F and *tet*O-R [28]; BlaOXA-61-F and BlaOXA-61-R, *cme*B-F and *cme*B-R, *aph*A-3-1-F and *aph*A-3-1-R [20]; and *gyr*A-F and *gyr*A-R [29]. The specificity of the primers was assayed by subjecting the sequences to basic nucleotide BLAST at NCBI (National Centre for Biotechnology Information; https://www.ncbi.nlm.nih.gov). The primers used were synthesized and purchased from Inqaba Biotechnologies (Pretoria, South Africa).

Cryopreserved DNA was defrosted and then amplified in a final reaction volume of 25 *μ*L in a BIO-RAD, T100™ Thermal Cycler (Singapore). The reaction mixture contained 12.5 *μ*l of OneTaq® 2X PCR Master Mix (New England Biolabs), 0.2 *μ*l of each forward and reverse primer, 5 *μ*l of template DNA, and the rest topped up with nuclease free water (BioConcept). Multiplex PCR (m-PCR) conditions for *tet* (O), *aph-3-1*, *cme*B, and *bla*OXA-61 consisted of an initial primary denaturation for 5 minutes at 94°C, a further 39 cycles of secondary denaturation at 94°C for 30 seconds, annealing at 54°C for 45 seconds, extension at 72°C for 1 minute, and final extension at 72°C for 10 minutes [11]. The amplification conditions for the *gyrA* gene (a 235-bp product) were as follows: an initial primary denaturation at 95°C for 5 minutes, 30 cycles at 95°C for 50 seconds, annealing at 53°C for 30 seconds, and 72°C for 1 min, followed by a final extension at 72°C for 7 minutes [30].

DNAse/RNAse free water (BioConcept) was used as a negative control. The amplicons were resolved by electrophoresis on a 1.5% agarose gel stained with ethidium bromide in Tris-Borate-EDTA (TBE) buffer; run at 60 V for 60 minutes, and then, visualised under ultraviolet light using the GelMax® 125 imager (UVP, Cambridge UK).

### 2.7. DNA Sequencing

A representative of positive amplicons (two *C. jejuni* and one *C. coli* for each antimicrobial resistance gene) generated with each primer was purified using QIAquick PCR Purification Kit (Qiagen) and commercially Sanger-sequenced in both directions at Inqaba Biotechnologies, Pretoria, South Africa. The forward and reverse sequences were edited, aligned, and assembled in consensus sequences using BioEdit software. Nucleotide sequences were subjected to BLASTn search tool (https://www.ncbi.nlm.nih.gov/BLAST), for confirmation of genes detected.

### 2.8. Data Handling and Analysis

Data were analyzed with statistical software R version 3.6.1. The difference was significant when *p* < 0.05. Cohen's kappa coefficient was used to assess the concordance between phenotypic antibiotic susceptibility and genotypic expression of resistance genes. According to McHugh [31], a kappa value of 0–0.2 indicates nonagreement, 0.21–0.39 (minimal level of agreement), 0.4–0.59 (weak level of agreement), 0.60–0.79 (moderate level of agreement), 0.80–0.90 (strong level of agreement), and above 0.90 (almost perfect level of agreement). A kappa value of 1 (100%) indicates total concordance between the two antibiotic susceptibility tests. The correlation between AMU and the occurrence of resistance was determined by Pearson's correlation coefficient (*r*) method. Furthermore, a 95% confidence interval was also determined for antibiotic resistance rates. All analyses were considered statistically significant at *P* < 0.05.

## 3. Results

### 3.1. Animal Health-Seeking Behaviour and Antimicrobial Use among Farmers in Kajiado County

When animals (cattle/chicken) were sick, majority of farmers (56.4%, 31/55) treated their animals themselves, 43.6% (24/55) sought services from a veterinarian or animal health assistant and/or community-based animal health workers. Those who self-treated their animal sought information on antimicrobial use from other farmers and agro-vet owners. The most commonly reported diseases in cattle prior 6 months prior to the study were as follows: mastitis (21/55, 38.2%), foot and mouth disease (14/55, 25.5%), contagious bovine pleuropneumonia (12/55, 21.8%), east coast fever (11/55, 20%), anaplasmosis (6/55, 10.9%), and lumpy skin disease (2/55, 3.6%). Clinical syndromes such as diarrhoea and abortion were also common in 16/55 (29.1%) and (12/55, 21.8%) of the farms, respectively. In poultry, most farms generally reported sick-bird syndromes such as ruffled feathers and/or dropping of wings, anorexia, diarrhoea, head tucked under wing, squinting or half-closed eyes, and solemnness of unknown cause. Seventy-five percent (41/55) of the farm owners interviewed were not aware/incognizant of the failing trend in antimicrobial therapy response.

Based on recall of antibiotic use in the last 6 months, 76.4% (42/55) of the farmers reported that they had used antibiotics mainly for treatment and prevention. Tetracyclines, aminoglycosides (streptomycin and gentamicin), and *β*-lactams-based antibiotics were the most commonly used antimicrobials to treat sick cattle and/or chicken (Table 1; Supplementary Figure S1). Antimicrobial use was generally higher in chicken production systems than in cattle for most of antibiotics apart from aminoglycosides and *β*-lactams (penicillins).

Some farmers (10/55, 18.2%) indicated using nonconventional medications such as herbs like *Aloe vera*, leaves of *Tithonia diversifolia* (Supplementary Figure S2), and chilli pepper among other “*mitishamba*” and/or “*dawa za kienyeji*” to relieve respiratory distress, diarrhoea, and other related sick-bird syndrome cases in chicken.

### 3.2. Antibiogram Profile of *C. jejuni* and *C. coli*

**Figure 2:**
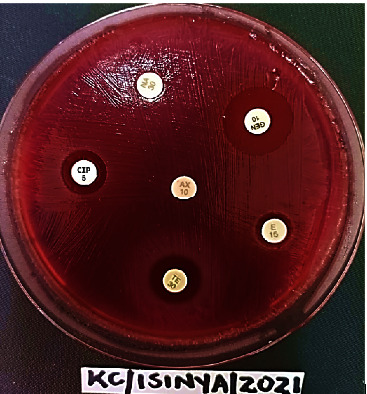
A representative photograph of antibiotic susceptibility profile of thermophilic *Campylobacter* isolate on Mueller–Hinton blood agar (MHBA) culture plate.

The test isolates showed varying degrees of inhibition zones to ampicillin (AMP), tetracycline (TE), erythromycin (E), nalidixic acid (NA), gentamicin (GEN), and ciprofloxacin (CIP) on Mueller–Hinton blood agar (MHBA) plate (Figure 2). The diameters of the inhibition zones were construed as either susceptible (S) or resistant (R) using the CLSI breakpoint criterion [26, 27].

The findings of antimicrobial resistance phenotypes performed on 29 *C. coli* and 74 *C. jejuni* isolates are tabulated in Table 2. Overall, all the 103 *Campylobacter* species were resistant to ampicillin (100%), followed by resistance to tetracycline (97.1%) and erythromycin (75.7%), to moderate resistance to ciprofloxacin (63.1%). The least resistance was observed for gentamicin (11.7%).

As for *C. coli*, all the isolates were resistant to ampicillin (100%), followed by resistance to tetracycline (96.6%), erythromycin (93.1%), and ciprofloxacin (69%); few strains were resistant to nalidixic acid and gentamicin (each at 10.3%). Tetracycline resistance in *C. coli* was seen more frequently in isolates from chicken and water samples (each at 100%). Similarly, *C. coli* resistance ciprofloxacin was prevalent in isolates from water samples and chicken at 100% and 77.8%, respectively. *C. coli* isolates from chicken and cattle swabs showed the highest resistance to erythromycin at 100% and 93.8%, respectively. Although no resistance to gentamicin was observed in any of the *Campylobacter* isolates from water samples; *C. coli* isolates from chicken recorded a relatively high resistance to gentamicin at 22.2%.

Likewise, ampicillin resistance was the most prevalent in *C. jejuni*, with levels of 100%, followed by resistance to tetracycline (97.3%), erythromycin (68.9%), ciprofloxacin (60.8%), and nalidixic acid (45.9%); few strains were resistant to gentamicin (1.3%). *C. jejuni* isolates from chicken showed a high rate of resistance to tetracycline and ciprofloxacin with 100% and 83.3%, respectively. Conversely, *C. jejuni* from cattle were highly resistant to erythromycin (76.3%) and gentamicin (15.8%), whereas those from water samples were 100% resistant to tetracycline, 66.7% resistance to ciprofloxacin and 50% resistance to nalidixic acid.

### 3.3. Multiple Drug Resistance and Resistance Patterns of *C. coli* and *C. jejuni*

*Campylobacter* isolates that were resistant to three or more classes of antibacterial agents were designated multidrug resistant (MDR). Ninety-nine of 103 (96.1%) isolates (29 (100%) *C. coli* and 70 (94.6%) *C. jejuni*) displayed MDR. In addition, the highest MDR was found among chicken isolates, with 100% (*n* = 39) MDR, regardless of the drug tested and/or *Campylobacter* species. Overall, a total of 14 different multiple drug resistance profiles were exhibited by *Campylobacter* species from cattle, chicken, and water samples are shown in Table 3. The most frequent MDR profiles of the isolates from different sources were resistant to AX-TE-E-CIP (29.1%), AX-TE-NA-CIP (18.4%), and AX-TE-E (16.5%).

### 3.4. Correlation between the Use of Various Antimicrobials and the Phenotypic Resistance among *Campylobacter* Isolates

Pearson correlation demonstrated highly significant (*p* < 0.01) positive correlations between antimicrobial use at the farm level and the phenotypic antibiotic resistance profiles for various drugs investigated in this study (Table 4). The highest positive correlations exist between the usage of tetracycline and its resistance at 31.4%. Beta-lactams and macrolide use showed positive correlation with resistance to erythromycin at 29.6% and 25.6%, respectively.

### 3.5. Detection of Genes Conferring Resistance, and Concordance between Resistance Phenotypes and Genotypes

**Figure 3:**
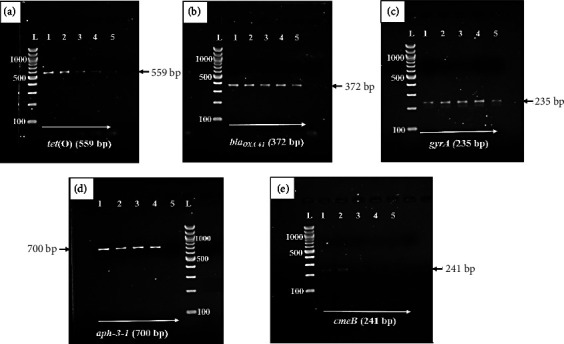
Exemplar of agarose gel electrophoresis of antimicrobial resistance genes: (L) 100 bp ladder/marker; 559 base pair (bp) *tet* (O) (a); 372 bp *bla*_*OXA-61*_ (b); 235 bp *gyrA* (c); 700 bp *aph*-3-1 (d); and 241 bp *cmeB* (e).

The occurrence of assayed genes conferring resistance to tetracyclines (*tet* (O)), *β*-lactams/ampicillin (*bla*_*OXA*-61_), aminoglycoside 3′-phosphotransferase gene (*aph*-3-1), fluoroquinolones (*gyrA*), and multidrug efflux pump (*cmeB*) were confirmed by PCR, by comparing the respective amplicon size with a 100 bp DNA marker (Figure 3).

**Figure 4:**
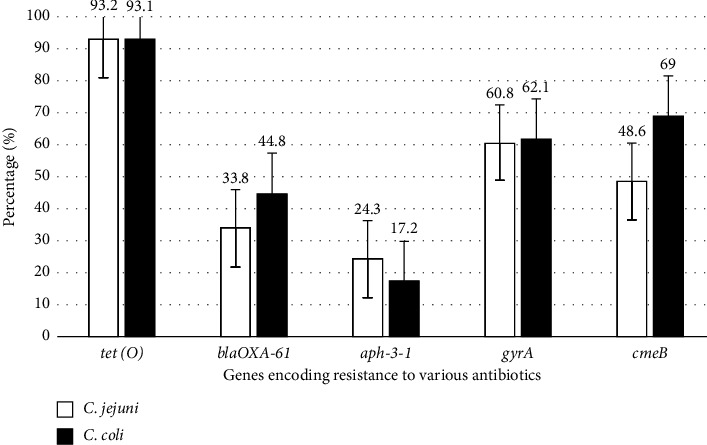
Percentage of *Campylobacter* isolates that harbour antimicrobial resistance gene. Results shown are the percentage prevalence ± standard error.

In general, the genes *tet* (O), *gyrA*, *cmeB*, bla_OXA*-61*_, and *aph*-3-1 were detected at 93.2%, 61.2%, 54.4%, 36.9%, and 22.3% of all *Campylobacter* isolates, respectively, irrespective of the source and *Campylobacter* species. The genes *tet* (O) (93.1% and 93.2%), *gyrA* (62.1% and 60.8%), *cmeB* (69% and 48.6%), bla_OXA*-61*_ (44.8% and 33.8%), and *aph*-3-1 (17.2% and 24.3%) were detected in *C. coli* and *C. jejuni* isolates, respectively.  Figure 4 illustrates the findings which indicate that *C. coli* isolates, as well as *C. jejuni* isolates, demonstrated more or less similar occurrence of antimicrobial resistance genes.

### 3.6. Comparison of Phenotypic and Genotypic Resistance to Antibacterial Agents

The highest correlations were found between the tetracycline resistance gene (tet (O)) and tetracycline-resistant phenotypes for *C. coli* (96.4%) and *C. jejuni* (95.8%) (Table 5). The findings showed significant associations (*p* < 0.05) among tetracycline, gentamicin, and ciprofloxacin-resistant phenotypes and their corresponding resistance genes for *C. jejuni* and *C. coli*. Interestingly, few nalidixic acid-resistant phenotypes harboured the *gyrA* gene (27% *C. jejuni* and 3.4% *C. coli*), compared to ciprofloxacin-resistant phenotypes that harbour the *gyrA* gene (48.3% *C. jejuni* and 41.9% *C. coli*).

In addition, using the Cohen's kappa coefficient, a moderate level of concordance between Kirby–Bauer disk diffusion method (phenotypic assay) and PCR (genotypic assay) was observed for tetracycline in both *C. coli* (kappa coefficient = 0.65) and *C. jejuni* (kappa coefficient = 0.55), while nonagreement was noted for nalidixic acid in both *C. coli* (kappa coefficient = 0.10) and *C. jejuni* (kappa coefficient = −0.036) (Table 5).

### 3.7. GenBank Accession Numbers

The partial sequences for some of the isolates from this study have been deposited in the GenBank database and assigned accession numbers: OQ389471, OQ389472, and OQ389473 for the *gyrA* gene; OQ390085 and OQ390086 for *tet* (O) gene; OQ421183 and OQ421184 for *bla*_*OXA-*61_ gene. Consensus sequences obtained from *cmeB* and *aph-*3-1 genes were too short with many gaps and as such were rejected on submission to GenBank.

## 4. Discussion

The world is at the verge of tipping over due to the adverse effects of AMR; with the latter emerging and spreading at a rate that by far surpasses development of newer drugs. It is notable that macrolide-fluoroquinolone-resistant bacterial pathogens particularly *Campylobacter* spp., have increased dramatically [32]. Fluoroquinolones and macrolides are prescribed as the first priority drugs for the treatment of human campylobacteriosis, and as such, increasing resistance trends pose a public health hazard.

*Campylobacter* species are naturally resistant to *β*-lactam antibiotics, including ampicillin [11]. None of the *Campylobacter* isolates in this study were susceptible to ampicillin, translating into 100% “acquired” resistance. Previous studies in other African countries including Tanzania and Morocco have reported resistance rate to this antibiotic at 63% and 95.2%, respectively [11, 33]. The high ampicillin-resistant phenotypes in this study might be due to the reported usage of *β*-lactams (including amoxicillin or a combination of procaine penicillin and dihydrostreptomycin sulphate or cloxacillin and ampicillin) among farmers in the treatment of bacterial infections such as mastitis in cattle.

Tetracycline is relatively inexpensive and highly effective against a wide range of microorganism; thus, it has been frequently used in livestock husbandry practices [34]. Therefore, it is not surprising that more than 97% of the isolates (96.6% for *C. coli* and 97.3% for *C. jejuni*) in this study were resistant to tetracycline. The results found in this study are comparable to a study conducted recently in various Kenyan counties, including Kajiado County [14]. Beyond Kenya, similar findings were reported in studies carried out in Spain [35], Tunisia [36], South Korea [37], and China [38].

Furthermore, the results demonstrated that the resistance rate among *Campylobacter* isolates recovered from livestock and water samples to erythromycin was 75.7%, including 93.1% for *C. coli* and 68.9% for *C. jejuni*. This resistance rate is somewhat worrying in contrast to previous findings from the outskirts of Thika, a city in Central Kenya [12]. The finding is consistent with the study by Asmai et al. [33] who also reported a high phenotypic *Campylobacter* resistance rate of 92.8% to erythromycin. Going by the findings of this study, macrolide (erythromycin) would no longer be considered as an alternative therapy in systemic campylobacter infections in man.

Ciprofloxacin, a fluoroquinolone, is one of the first line antibiotics in the treatment of clinical campylobacteriosis in man. Notably, significant 63.1% ciprofloxacin-resistant isolates (69% *C. coli* and 60.8% *C. jejuni)* compared to strains resistant to nalidixic acid at 35.9% (10.3% *C. coli* and 45.9% *C. jejuni*) were reported in this study. The observed resistance to ciprofloxacin is comparable to other studies in Kenya [12], Ethiopia [39], and Poland [40]. The relatively low resistance to nalidixic acid observed in this study is in contrast with those on *Campylobacter* isolates from backyard chicken in Central Kenya, where resistance to nalidixic acid was observed at 77.4% [12]. The level of resistance to nalidixic acid observed in this study is however concordant with findings found in studies from other regions: Poland [41], Tanzania [7], South Africa [42], and the USA [43]. The low resistance to nalidixic acid may be as a result of a decrease in the use of quinolones including nalidixic acid, over most sought-after fluoroquinolones (such as ciprofloxacin) for curative or prophylactic purposes.

The overall resistance for gentamicin was low (11.7%) with *C. jejuni* isolates portraying slightly higher (12.2%) resistance than *C. coli* (10.3%). The findings concord with reports from other African and European states. For instance, in Tanzania, 11.8% of the *Campylobacter* isolates from dressed beef carcasses and raw milk in Tanzania were resistant to gentamicin [11]. In North African countries such as Morocco, 7.1% of the isolates from poultry were gentamycin-resistant [33]. Low resistance to gentamicin was also been observed in Spain, where 12.1% and 14.7% of *C. coli* strains from cattle and broilers were resistant [35]. The relatively low resistance could possibly be due to restricted use for systemic infections [44], and also due to the fact that there are no oral formulations to be administered in drinking water or feeds for use in livestock production.

However, the results of phenotypic and genotypic assays of resistance to various antibiotics were partially concordant; moderate level of agreement being observed only in tetracycline. A similar observation was also reported by Kashoma et al. [11]. This deduces that other factors beyond this study, including the occurrence of other molecular determinants that encode resistance could be involved.

The *tet* (O) gene is the most common ribosomal protection mechanism mediating *Campylobacter* resistance to tetracycline. However, other genes such as *tet* (A), *tet* (K), *tet* (B), and multidrug efflux, have also been reported. Almost all the tetracycline-resistant phenotypes were shown to harbour the *tet* (O) gene at 93.1%. This is higher in this study than the percentage of the same gene in chicken samples in a report by Nguyen et al. [12]. However, similar results to this study have been reported in China [38].

The *gyrA* gene was confirmed in 61.2% of the isolates, including 62.1% *C. coli* and 60.8% *C. jejuni* in this study. The substitution of threonine to isoleucine (Thr86Ile region) in the *gyrA* genome confers cross-resistance to both quinolones (nalidixic acid) and fluoroquinolones (ciprofloxacin). However, Ge et al. [45] reported upper-level resistance to ciprofloxacin linked to a mutation in the Thr86Ile region of the *gyrA* genome. The results of this study further revealed that low nalidixic acid-resistant phenotypes possessing *gyrA* genome compared to the ciprofloxacin-resistant phenotypes possessing *gyrA* genome. The discrepancies in the *gyrA* gene detection rate for ciprofloxacin and nalidixic acid resistance could further be explained by the fact that occurrence of point mutation in the Thr86Ala region of the gyrase subunit A gene (by substitution of threonine to alanine) has been linked with high nalidixic acid-resistant and low ciprofloxacin-resistant *C. jejuni* [45]. Indeed, more molecular studies are needed to explore *gyrA* gene sequences and other antibiotic resistance genes incriminated in *Campylobacter* spp. resistance to nalidixic acid and ciprofloxacin.

The *cme*B gene, conferring resistance to multiple antibiotics including macrolides (erythromycin), *β*-lactams (ampicillin), tetracyclines, and fluoroquinolones (ciprofloxacin) was detected in over 54% of the isolates (69% *C. coli* and 48.6% *C. jejuni*). However, the findings of this study are much lower than previous reports in Tunisia [36].

Despite the high resistance to ampicillin reported in this study, *β*-lactam conferring gene (*bla*_*OXA*-61_) was detected in only 36.9% of all *Campylobacter* isolates (44.8% in *C. coli* and 33.8% in *C. jejuni*), suggesting that other means of acquired ampicillin resistance could be involved. Comparable findings were reported by Kashoma et al. [11], where 52.6% and 28.1% of *C. coli* and *C. jejuni* strains, respectively, were found to harbour the *bla*_*OXA*-61_ gene. Undeniably, other genetic determinants including modifications in outer membrane porins and/or decreased affinity of penicillin-binding protein (PBP) and efflux pump are most likely involved [11, 46].

More than 22% of the strains were found to possess the *aph*-3-1 gene. Obviously, gentamicin-resistant phenotypes cannot be elucidated by *aph*-3-1 gene. However, our findings were much higher than previous reports in Africa [11]. Yet Hailu et al. [43] reported 100% detection rate amongst *Campylobacter* isolates from dairy cattle and chicken manure in the USA.

Multidrug resistance (MDR) presents a public health threat by limiting antibacterial agents to choose from for curative therapy. Almost all the *Campylobacter* isolates (>96%) in this study were resistant to three or more of the six tested antibacterial agents; with *C. coli* and *C. jejuni* reported 100% and 94.6% MDR, respectively. Ampicillin-tetracycline-erythromycin-ciprofloxacin (AX-TE-E-CIP) and AX-TE-NA-CIP were the most common MDR patterns in both *C. coli* and *C. jejuni*. The MDR rate reported in this study is much higher than what has been reported in some European nations; for instance, in Poland, where MDR for *Campylobacter* isolates from raw chicken meat was 7% [40]. However, the findings of this study are concordant with some studies in other African countries: 95% of the *Campylobacter* isolates from broiler in Morocco displayed drug resistance to ≥3 drugs [33]; 95.5% of isolates from livestock (cattle and shoat), poultry, human, and water in Ethiopia [39]; 94.7% of the strains from poultry in Ghana [47], and 32.5% of in *Campylobacter* isolates from beef cattle in South Africa [42]. The observed discrepancies in MDR in *Campylobacter* may possibly be explained by the following: (1) level of intensification and type of production system; (2) the introduction and implementation of legislation to minimize antimicrobial use in livestock in European countries. In underdeveloped nations including Kenya, there are laws and rules on antimicrobials use in food animals; however, enforcement is done to a limited extent or practically nonexistent. Consequently, higher resistances to most antimicrobial agents tested may be due to the relatively unrestricted use of antimicrobial agents in animal treatment that is practiced in most of the developing countries [48]. In this study, extensive use of antimicrobial drugs was observed in this study, with tetracyclines, aminoglycosides, and *β*-lactams being commonly used. Excessive use of these antibiotics in livestock has also been reported in other studies [49, 50]. Moreover, antibiotic usage was positively correlated with the high level of resistance to tetracyclines and erythromycin amongst *Campylobacter* isolates in this study.

In this study, extensive misuse of antimicrobials was observed in this study, where 56.4% of farmers treated their animals themselves without the prescription or advice from a qualified veterinarian. This finding agrees with Chepkwony [13], who reported that 67.5% of livestock owners admitted injecting drugs into their animals themselves without professional consultation. Although the self-reported use of antibiotics among farmers in this study precluded establishment of diagnosis and dosage regime; there is a possibility that antibiotics are often administered in absence of a confirmatory diagnosis, or antibiotic susceptibility testing in response to various clinical syndromes or illnesses, some of which are caused by nonbacterial pathogens such as foot and mouth disease, lumpy skin disease, or tick-borne diseases. Therefore, inadequate veterinary skills and accessibility is of great concern and could accelerate antibiotic overuse in livestock; thus, they may be linked with the evolution of MDR *Campylobacter* isolates in the county.

Finally, where the use of fluoroquinolones among other antibiotics in food production is banned, the frequency of *Campylobacter*-resistant isolates is relatively low. For instance, Australia, where administration of fluoroquinolones in food animals is prohibited, recorded *Campylobacter* strains susceptible to ciprofloxacin recovered from pigs in 2004 [51]. However, years later, fluoroquinolone-resistant *Campylobacter* isolates emerged and were detected among Australian chickens, even in the absence of fluoroquinolone application [52]. These fluoroquinolone-resistant *Campylobacter* isolates might have emerged from outside and brought into Australian chicken by people, vectors, or wild birds [52]. These findings dramatically underline the critical role of biosecurity in the overall fight against antimicrobial resistance. Consequently, even as nations call for a policy on minimizing application of antimicrobials in livestock; stringent farm biosecurity measures come handy in the overall fight against antimicrobial resistance.

## 5. Conclusions

In this study, extensive use of antimicrobial drugs was observed in this study, with tetracyclines, aminoglycosides, and *β*-lactams being commonly used. Application of antibiotics in cattle and poultry production systems was positively correlated with the high level of resistance to tetracyclines and erythromycin. This highlights the significance of the warranted application of antibacterial agents in the said production systems in the county. Regarding antimicrobial resistance, almost all isolates (96.1%) displayed MDR, with *C. coli* expressed greater resistance to three or more of the assayed antimicrobials. This might further limit treatment options for *Campylobacter* infections. A high level of resistance to ampicillin, tetracycline, erythromycin, and ciprofloxacin was found among the *Campylobacter* strains. As such, none of the priority drugs in *Campylobacter* infections therapy can be prescribed in the county. Chicken-derived *Campylobacter* strains showed greater resistance; this could be due to the widespread use of antibiotics in the poultry production system compared to the cattle production system. The *tet* (O), *gyrA,* and *cmeB* were the most frequently detected genes, while the occurrence of bla_OXA*-61*_ and *aph*-3-1 was significantly lower (*p* < 0.05).

## 6. Recommendations

Furthermore, molecular studies should include all the cryptic antibiotic resistance genes and plasmids in *C. jejuni* and *C. coli* strains to give insights on their transmission and possible transfer to other *Campylobacter* strains. The existing national action plan on AMR spearheaded by the ministries of health and agriculture, livestock, and fisheries in Kenya must strengthen the surveillance programs and policies advocating for a reduction in unwarranted use of antibiotics. Moreover, the veterinary directorate at the county and national governments ought to be on the fore-front in managing and implementing appropriate biosecurity measures aimed at fighting antimicrobial resistance. Screening of alternative treatment, e.g., use of medicinal plant extracts (*Aloe vera*, *Tithonia diversifolia*, and chilli pepper) needs to be encouraged, in effort to reduce usage of antibiotics.

## Figures and Tables

**Table 1:** Antibiotics commonly used by farmers for the treatment of sick chicken and cattle in Kajiado County, Kenya.

Antibiotic classes	Trade name(s)	Active ingredient(s)	Animal uses	Proportions of farms using the drugs
Tetracyclines	Aliseryl™	Oxytetracycline, erythromycin, streptomycin, colistin, and vitamins	Chicken	39/42
	Tylodoxy 200™	Doxycline and tylosin	Chicken	
	Vetoxy™	Oxytetracycline	Chicken	
	Tetracolivit™	Oxytetracycline, colistin, and vitamins	Chicken	
	Ulticycline 10%™/oxytetra 10%™/oxymet-10/adamycin 10%™/alamycinLA 300™/twigamycin™	Oxytetracycline	Cattle	

Aminoglycosides	Pen & strep™/penistrep™	Dihydrostreptomycin sulphate and procaine penicillin	Cattle	24/42
	Vetgenta™	Gentamicin	Cattle	
	Terrexine^TM^	Kanamycin sulphate and cephalexin	Cattle	
	Gentamast™	Gentamicin	Cattle	
	Aliseryl™	Streptomycin, oxytetracycline, erythromycin, colistin, and vitamins	Chicken	

*β*-Lactams (penicillins)	Bimoxyl LA™	Amoxicillin	Cattle	19/42
	Bovaclox^TM^	Cloxacillin and ampicillin	Cattle	
	Pen & strep™/penistrep™	Procaine penicillin and dihydrostreptomycin sulphate	Cattle	

Macrolides	FosBac™/fostyl plus ^TM^	Tylosin sulphate and calcium fostomycin	Chicken	11/42
	Tylodoxy 200™	Tylosin tartrate and doxycline	Chicken	
	Marolan WS	Tylosin tartrate	Chicken	
	Tylosin injection	Tylosin tartrate	Cattle	
	Aliseryl™	Erythromycin, streptomycin, oxytetracycline, colistin, and vitamins	Chicken	

Sulphonamides	BIOSOL™/TRIMOVET™	Trimethoprim-sulfamethoxazole	Chicken	9/42
	Esb3™	Sulphachloropyrazine	Chicken	
	Diseptoprim^TM^	Trimethoprim-sulfamethoxazole	Cattle	

Polymyxins	Colistin sulphate	Colistin sulphate		9/42
	Tetracolivit™	Oxytetracycline, colistin, and vitamins		
	Aliseryl™	Erythromycin, streptomycin, oxytetracycline, colistin, and vitamins	Chicken	

Cephalosporins	Terrexine^TM^	Kanamycin sulphate and cephalexin	Cattle	3/42

**Table 2:** Phenotypic antimicrobial resistance profiles for *C*. *coli* and *C*. *jejuni* isolates.

Antimicrobial agents	No. of resistant isolates (%)	*Source and number of isolates showing resistance (%)*							
		*C. coli* (*N* = 29)				*C. jejuni* (*N* = 74)			
		Cattle	Chicken	Water	Total	Cattle	Chicken	Water	Total
Ampicillin	103 (100)	16 (100)	9 (100)	4 (100)	29 (100)	38 (100)	30 (100)	6 (100)	6 (100)
Tetracycline	100 (97.1)	15 (93.8)	9 (100)	4 (100)	28 (96.6)	36 (94.7)	30 (100)	6 (100)	72 (97.3)
Gentamicin	12 (11.7)	1 (6.3)	2 (22.2)	0	3 (10.3)	6 (15.8)	3 (10)	0	9 (12.2)
Erythromycin	78 (75.7)	15 (93.8)	9 (100)	3 (75)	27 (93.1)	29 (76.3)	19 (63.3)	3 (50)	51 (68.9)
Ciprofloxacin	65 (63.1)	9 (56.3)	7 (77.8)	4 (100)	20 (69)	16 (42.1)	25 (83.3)	4 (66.7)	45 (60.8)
Nalidixic acid	37 (35.9)	1 (6.3)	1 (11.1)	1 (25)	3 (10.3)	15 (39.5)	16 (53.3)	3 (50)	34 (45.9)

**Table 3:** Resistance patterns of *C. jejuni* and *C. coli* isolated from cattle, chicken, and water samples.

Antimicrobial resistance patterns	No. of resistant isolates (%)	*Source and number of isolates showing resistance (%)*							
		*C. coli* (*N* = 29)				*C. jejuni* (*N* = 74)			
		Cattle (*n* = 16)	Chicken (*n* = 9)	Water samples (*n* = 4)	Total	Cattle (*n* = 38)	Chicken (*n* = 30)	Water samples (*n* = 6)	Total
AX-TE	2 (1.9)	0	0	0	0	2 (5.3)	0	0	2 (2.7)
AX-E	2 (1.9)	0	0	0	0	1 (2.6)	0	1 (16.7)	2 (2.7)
AX-TE-E	17 (16.5)	7 (43.8)	2 (22.2)	0	9 (31)	6 (15.8)	1 (3.3)	1 (16.7)	8 (10.8)
AX-TE-CIP	4 (3.9)	1 (6.3)	0	1 (25)	2 (6.9)	0	1 (3.3)	1 (16.7)	2 (2.7)
AX-E-CIP	1 (1)	1 (6.3)	0	0	1 (3.4)	0	0	0	0
AX-E-NA	1 (1)	0	0	0	0	1 (2.6)	0	0	1 (1.4)
AX-TE-E-NA	9 (8.7)	0	0	0	0	5 (13.2)	4 (13.3)	0	9 (12.2)
AX-TE-E-CIP	30 (29.1)	5 (31.3)	4 (44.4)	2 (50)	11 (37.9)	9 (23.7)	10 (33.3)	0	19 (25.7)
AX-TE-NA-CIP	19 (18.4)	0	0	0	0	7 (18.4)	10 (33.3)	2 (33.3)	19 (25.7)
AX-TE-E-GEN	3 (2.9)	0	0	0	0	3 (7.9)	0	0	3 (4.1)
AX-TE-E-NA-CIP	6 (5.8)	1 (6.3)	1 (11.1)	1 (25)	3 (10.3)	1 (2.6)	1 (3.3)	1 (16.7)	3 (4.1)
AX-TE-E-NA-GEN	1 (1)	0	0	0	0	1 (2.6)	0	0	1 (1.4)
AX-TE-E-GEN-CIP	7 (6.8)	1 (6.3)	2 (22.2)	0	3 (10.3)	2 (5.3)	2 (6.7)	0	4 (5.4)
AX-TE-E-NA-GEN-CIP	1 (1)	0	0	0	0	0	1 (3.3)	0	1 (1.4)

**Table 4:** Pearson correlation between antibiotic use at farm level and occurrence of resistance.

Antimicrobial usage at farm levels	Comparisons	*Phenotypic resistance using the disk diffusion methods*				
		TE	E	NA	GEN	CIP
Tetracycline usage	Pearson correlation (R)	0.314^*∗∗*^	0.006	−0.110	0.131	−0.085
	Sig. (2-tailed)	0.001	0.950	0.270	0.190	0.397

Aminoglycoside usage	Pearson correlation	−0.053	0.099	−0.032	0.150	−0.033
	Sig. (2-tailed)	0.613	0.345	0.759	0.149	0.75

Macrolide usage	Pearson correlation	0.022	0.256^*∗*^	−0.199	−0.022	−0.01
	Sig. (2-tailed)	0.830	0.013	0.055	0.831	0.923

*β*-lactam usage	Pearson correlation	−0.106	0.296^*∗∗*^	−0.138	0.054	−0.163
	Sig. (2-tailed)	0.309	0.004	0.186	0.608	0.117

Sig.: significance; ^*∗∗*^Correlation is significant at the 0.01 level (2-tailed); ^*∗*^Correlation is significant at the 0.05 level (2-tailed).

**Table 5:** Correlations between phenotypic and genotypic resistance among *Campylobacter* isolates.

Drug tested phenotypically using disc diffusion method	Antimicrobial resistance gene detected using PCR method	*Campylobacter*species	No. of isolates with resistant phenotype (%)	No. of isolates possessing resistance genes or mutations corresponding to resistance phenotype (%)	Correlation between genotypes and phenotype (%)	*Measure of agreement between the methods*
						Kappa value
Ampicillin	*bla*_*OXA-61*_	*C. coli*	29 (100)	13 (44.8)	44.8	
		*C. jejuni*	74 (100)	25 (33.8)	33.8	

Tetracycline	*tet*(O)	*C. coli*	28 (96.6)	27 (93.1)	96.4	0.65^*∗*^
		*C. jejuni*	72 (97.3)	69 (93.2)	95.8	0.55^*∗*^

Gentamicin	*aph*-3-1	*C. coli*	3 (10.3)	2 (6.9)	66.7	0.43
		*C. jejuni*	9 (12.2)	8 (10.8)	88.9	0.51

Erythromycin	—	*C. coli*	27 (93.1)	—	—	—
		*C. jejuni*	51 (68.9)	—	—	—

Ciprofloxacin	*gyrA*	*C. coli*	20 (69)	14 (48.3)	70	0.24
		*C. jejuni*	45 (60.8)	31 (41.9)	68.9	0.21
Nalidixic acid		*C. coli*	3 (10.3)	1 (3.4)	33.3	−0.10
		*C. jejuni*	34 (45.9)	20 (27)	58.8	−0.04

## Data Availability

All the data relating to this study are available on mail request to the corresponding author.

## Supplementary Materials

Supplementary Materials Field observations supporting animal health-seeking behaviour and antimicrobial use among farmers in Kajiado County was submitted as a supplementary file. Supplementary Figure S1: drugs commonly used in chicken and cattle production systems by small-scale farmers in Kajiado County, Kenya. Supplementary Figure 2: herbal decoction from *Tithonia diversifolia* leaves used against sick-bird syndromes in chicken production systems in Kajiado County.
