# Supplementary material for: Antimicrobial Usage, Susceptibility Profiles, and Resistance Genes in Campylobacter Isolated from Cattle, Chicken, and Water Samples in Kajiado County, Kenya
Source: Int J Microbiol. 2023 Mar 22;2023:8394605. doi: 10.1155/2023/8394605 (PMC10060070; doi:10.1155/2023/8394605)
Supplement: Supplementary Materials — Field observations supporting animal health-seeking behaviour and antimicrobial use among farmers in Kajiado County was submitted as a supplementary file. Supplementary Figure S1: drugs commonly used in chicken and cattle production systems by small-scale farmers in Kajiado County, Kenya. Supplementary Figure 2: herbal decoction from Tithonia diversifolia leaves used against sick-bird syndromes in chicken production systems in Kajiado County. [file 8394605.f1.zip › Supplementary Figure S1.docx]

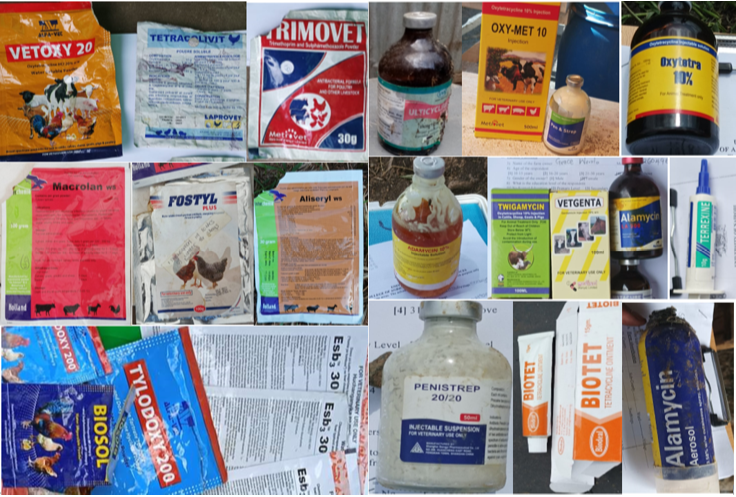


**Supplementary Figure S1: Drugs commonly used in chicken and cattle production systems by small-scale farmers in Kajiado County, Kenya**
